# Most Do, but Some Do Not: CD4^+^CD25^−^ T Cells, but Not CD4^+^CD25^+^ Treg Cells, Are Cytolytic When Redirected by a Chimeric Antigen Receptor (CAR)

**DOI:** 10.3390/cancers9090112

**Published:** 2017-08-29

**Authors:** Andreas A. Hombach, Hinrich Abken

**Affiliations:** 1 Department I Internal Medicine, University Hospital Cologne, Cologne D-50931, Germany; andreas.hombach@uk-koeln.de; 2 Center for Molecular Medicine Cologne, University of Cologne, Robert-Koch-Str. 21, Cologne D-50931, Germany

**Keywords:** CAR, chimeric antigen receptor, T cell, CD4, Treg cell

## Abstract

Evidences are accumulating that CD4^+^ T cells can physiologically mediate antigen specific target cell lysis. By circumventing major histocompatibility complex (MHC)-restrictions through an engineered chimeric antigen receptor (CAR), CD4^+^ T cells lyse defined target cells as efficiently as do CD8^+^ T cells. However, the cytolytic capacity of redirected CD4^+^CD25^−^ T cells, in comparison with CD4^+^CD25^+^ regulatory T (Treg) cells was so far not thoroughly defined. Treg cells require a strong CD28 signal together with CD3ζ for activation. We consequently used a CAR with combined CD28­CD3ζ signalling for redirecting CD4^+^CD25^−^ T cells and CD4^+^CD25^+^ Treg cells from the same donor. CAR redirected activation of these T cell subsets and induced a distinct cytokine pattern with high IL-10 and a lack of IL-2 release by Treg cells. Despite strong antigen-specific activation, CAR Treg cells produced only weak target cell lysis, whereas CD4^+^CD25^−^ CAR T cells were potent killers. Cytolysis did not correlate with the target cell sensitivity to Fas/FasL mediated killing; CD4^+^CD25^−^ T cells upregulated perforin and granzyme B upon CAR activation, whereas Treg cells did less. The different cytolytic capacities of CAR redirected conventional CD4^+^ cells and Treg cells imply their use for different purposes in cell therapy.

## 1. Introduction

T cells can be antigen-specifically redirected by genetic engineering with a chimeric antigen receptor (CAR) that combines the advantages of antibody mediated target recognition with the induction of T cell effector functions [1]. Upon antigen engagement, CAR T cells execute a cellular response in a specific fashion, as indicated by cytokine release, T cell amplification and lysis of cognate target cells. Since the CAR circumvents the MHC (major histocompatibility complex) restriction, both CD8^+^ and CD4^+^ T cells can be activated through the same CAR. CD4^+^ T cells are of particular interest for the use in adoptive cell therapy of cancer because they differentiate into various cell subsets which initiate, modulate, or suppress a cellular and humoral immune response [2]. CD4^+^ T cells represent the majority in number of the peripheral blood, and, in addition to CD8^+^ cytotoxic T cells, can exhibit cytolytic capacities. The recently described MHC class II-restricted CD4^+^ cytotoxic T cells (CD4^+^ CTLs) emerge during chronic viral infections [3,4] and during an anti-tumor response [5,6]. The physiologic function of cytotoxic CD4^+^ CTLs is not yet completely understood [7]. On the other hand, CD4^+^ regulatory T (Treg) cells which predominantly display a CD4^+^CD25^high^ phenotype [8] suppress ongoing immune reactions through multiple mechanisms, potentially also through CTL like properties [8,9]. The situation raises the question of whether CD4^+^CD25^high^ Treg cells can mediate MHC unrestricted, but antigen specific cytolysis when redirected by a CAR as do CAR redirected CD4^+^CD25^−^ T cells.

To explore the CD4^+^ T cell mediated target cell lysis upon CAR activation, we isolated CD4^+^CD25^−^ and CD4^+^CD25^high^ T cells from the peripheral blood of healthy donors and engineered the cells with a CD28-CD3ζ signaling CAR with specificity for the carcinoembryonic antigen (CEA) [10]. Combined CD28-CD3ζ, signaling by the “second generation”, CAR is required since in particular Treg cells depend on a strong CD28 signal along with CD3/TCR signaling for activation and amplification [11]. In contrast to the “first generation” CAR, which signals only through the primary CD3ζ signal, the “second generation” CAR provides the primary signal together with a costimulatory signal through the fused intracellular signaling domains like CD28-CD3ζ [10,12]. Recording CAR redirected lysis in a thoroughly controlled side-by-side comparison revealed that both CD4^+^ T cell subsets have different capacities for killing; CAR redirected CD4^+^CD25^+^ regulatory T cells have low, if any, cytotoxic activity indicating that the target cell lysis is not a major function of these cells, whereas CAR redirected CD4^+^CD25^−^ T cells are potent target cell killers. Due to their different cytolytic capacities the T cell subsets are suitable for different purposes in the adoptive cell therapy of various diseases.

## 2. Materials and Methods

### 2.1. Antibodies and Reagents

The anti-idiotypic monoclonal antibody (mAb) BW2064/36 with specificity for the anti-CEA mAb BW431/26 was described earlier [13]. The hybridoma cell line OKT3, that produces the anti-CD3 mAb OKT3, was obtained from ATCC, Rockville, MD, USA (ATCC CRL 8001). The agonistic anti-CD28 antibody 15E8 was kindly provided by R. van Lier (Academic Medical Center, Amsterdam, The Netherlands). MAbs were affinity purified from supernatants utilizing an agarose immobilized goat anti-mouse IgG1 (Sigma, Deisenhofen, Germany) or a sepharose (Amersham Pharmacia, Freiburg, Germany) immobilized goat anti-mouse IgG2a antibody (Southern Biotechnology, Birmingham, AL, USA). The fluorescein-isothiocyanate (FITC)-conjugated anti-CD4 and anti-CD3 mAbs, respectively, were purchased from Dako, Hamburg, Germany. The PE-conjugated anti-CD25 mAb was purchased from Miltenyi Biotec, Bergisch Gladbach, Germany. PE-conjugated antibodies for intracellular staining of perforin and granzyme B were purchased from BD Bioscience Heidelberg, Germany. Matched pair antibodies for detection of human IFN-γ, IL-2 and IL-10, respectively, were purchased from BD Bioscience.

### 2.2. Cell Lines

HEK 293T cells are human embryonic kidney cells that express the SV40 large T antigen [14]. SW948 (ATCC CLL 237 H508 (ATCC CCL 253), and LS174T (ATCC CCL 188) are CEA-expressing colon carcinoma cell lines, Colo320 (ATCC CCL 220.1) is a CEA-negative cell line, Jurkat (ATCC TIB 152) is a Fas susceptible cell line. The CD30^+^ T lymphoma cell line Myla was kindly provided by Prof. R. Dummer, Zurich. The cell lines were cultured in RPMI 1640 or DMEM medium supplemented with 10% (*v*/*v*) FCS (all Life Technologies, Paisly, UK).

### 2.3. Magnetic Activated Cell Sorting (MACS)

Peripheral blood lymphocytes from healthy donors were isolated by density centrifugation, and monocytes were depleted by plastic adherence. Non-adherent lymphocytes were washed with cold phosphate buffered saline (PBS) containing 0.5% (*w*/*v*) BSA, 1% (*v*/*v*) FCS and 2 mM EDTA. CD4^+^CD25^+^ Treg cells and CD4^+^CD25^−^ T cells were isolated by magnetic activated cell sorting (MACS) utilizing the CD25 T cell isolation kit (Miltenyi) as recommended by the manufacturer. The number of isolated CD4^+^CD25^+^ Treg cells and of CD4^+^CD25^−^ T cells was determined by flow cytometry. Isolated T cells were washed, counted, and utilized for further experiments. For in vitro amplification of CD4^+^CD25^+^ T cells, Polysorb culture plates (Nunc, Roskilde, Denmark) were coated with OKT3 and 15E8 mAbs (each 5 µg/mL) over night in PBS, washed twice with PBS and isolated T cells were added in X-Vivo15 medium, 10% (*v*/*v*) FCS supplemented with IL-2 (1000 U/mL) (Endogen, Woburn, MA, USA) and IL-15 (1 ng/mL) (R&D Systems, Wiesbaden, Germany) based on our previous results [15,16,17]. CD4^+^CD25^−^ T cells were initially cultured in RPMI 1640 medium, 10% (*v*/*v*) FCS in the presence of OKT3 and 15E8 mAb (each 100 ng/mL) and IL-2 (1000 U/mL) (Endogen). After activation for 48 h CD4^+^CD25^−^ T cells were further cultivated in RPMI 1640 medium, 10% (*v*/*v*) FCS supplemented with IL-2 (400 U/mL).

### 2.4. CAR Engineering and Transduction of CD4^+^ T Cells

The CEA and CD30 specific second generation CARs BW431/26-scFv-Fc-CD28-CD3ζ and HRS3-scFv-Fc-CD28-CD3ζ were previously described [10,18]. Retroviral vector DNA was cotransfected with the retroviral helper plasmid DNAs pHIT60 and pCOLT [14] (each 1 µg DNA/10^5^ cells) into 293T cells utilizing Polyfect reagent (Qiagen, Hilden, Germany). MACS-isolated CD4^+^ T cells from the peripheral blood were activated by addition of IL-2 and OKT3 mAb as described above, washed, resuspended in RPMI 1640 medium with IL-2 (400 U/mL), and cocultivated for 48 h with transiently transfected HEK 293T cells. T cells were harvested and receptor expression was monitored by flow cytometry.

### 2.5. Immunofluorescence Analysis

CAR T cells were identified by two colour immunofluorescence utilizing PE-conjugated anti-human IgG1 (0.1 µg/mL) and FITC-conjugated anti-CD3 (5 µg/mL) antibodies. Labelled cells were analysed on a FACS Calibur cytofluorometer (BD Bioscience). Expression of cytolytic effector molecules was monitored as follows: CAR T cells were stained with an anti-CD4-FITC mAb and subsequently fixed and permeabilized utilizing the “cytofix/cytoperm” kit (BD Bioscience), as according to the manufacturer’s recommendations. Permeabilized cells were incubated with PE-conjugated anti-perforin, and anti-granzyme B antibodies, respectively, washed and analysed, as described above.

### 2.6. Death Receptor Mediated Apoptosis of Target Cells

Tomonitor the Fas sensitivity of cell lines, cells were incubated in 96-well microtiter plates with recombinant Fas-L (2 µg/mL) (Ancell, Bayport, MI, USA), for 24 h and the viability of cells was monitored by a XTT-(2,3-bis-(2-methoxy-4-nitro-5-sulfophenyl)-2H-tetrazolium-5-carboxanilide)—based colorimetric assay, as described below.

### 2.7. Suppressor Assay

CD3^+^ responder T cells (10^7^) were washed twice in PBS, incubated with carboxy-fluorescein diacetate succinimidyl ester (CFSE) labeling solution (1 µM final concentration) (InVitroGen, Karlsruhe, Germany) for 5 min on ice and washed five times with cold RPMI 1640 medium, 10% (*v*/*v*) FCS. CD4^+^CD25^−^ and CD4^+^CD25^+^ CAR T cells and non-modified T cells (each 5 × 10^4^ cells/well), respectively, were cocultivated with CFSE-labeled CD3^+^ responder T cells in the presence of soluble anti-CD3 mAb OKT3 (10 µg/mL) plus anti-CD28 mAb 15E8 (1 µg/mL) mAb. After five days of incubation, CFSE labeled cells were analyzed by flow cytometry. The numbers of cycling and non-cycling CFSE labeled T cells were determined.

### 2.8. Stimulation of CAR T Cells and Cytokine ELISAs

CD4^+^ CAR T cells were cocultivated in serial dilutions (1.25–10 × 10^4^ cells/well) with tumor cells (5 × 10^4^ cells/well) for 48 h in 96-well round bottom plates. Viability of target cells was recorded as described below and culture supernatants were analysed for secretion of IFN-g, IL-2, and IL-10 by CAR T cells by ELISA. Briefly, cytokines in the supernatant were bound to a solid phase anti-human IFN-g, IL-10, or IL-2 capture antibodies (each 1 µg/mL), and detected by a biotinylated anti-human IFN-g, IL-10 and IL-2 detection antibodies each 0.5 µg/mL), respectively. The reaction product was visualized by a peroxidase-streptavidin-conjugate (1:10,000) and ABTS^®^ (both from Roche Diagnostics) as substrate. The amount of cytokine was calculated by utilizing reference standard curves with known amounts of cytokines.

### 2.9. XTT-Based Cytotoxicity Assay

Specific cytotoxicity of receptor grafted CD4^+^ T cells against target cells was monitored by a XTT based colorimetric assay according to Jost et al. [19]. Briefly, CAR T cells and non-transduced T cells were cocultivated with target cells in triplicates in round-bottom microtiter plates for 48 h as described above XTT reagent (1 mg/mL) (“Cell Proliferation Kit II”, Roche Diagnostics) was added to the cells and incubated for 30–90 min at 37 °C. Reduction of XTT to formazan by viable target cells was monitored colorimetrically at an absorbance wavelength of 450 nm and a reference wavelength of 630 nm. Maximal reduction of XTT was determined as the mean of 6 wells containing tumour cells only, the background as the mean of 6 wells containing RPMI 1640 medium, 10% (*v*/*v*) FCS. The non-specific formation of formazan due to the presence of effector cells was determined from triplicate wells containing effector cells in the same number, as in the corresponding experimental wells. The viability of target cells [%] was calculated as follows: viability [%] = [OD_(exp. wells­corresponding number of effector cells)_]/[OD_(tumor cells without effector cells­medium)_] × 100; specific cytotoxicity [%] = 100 − viability [%].

### 2.10. Statistics

Experimental results from independent representative experiments are reported as mean values ± standard deviation (SD). Significant analyses were performed by Student’s *t*-test using Microsoft Excel and Graphpad Prism, respectively.

## 3. Results

**Figure 1:**
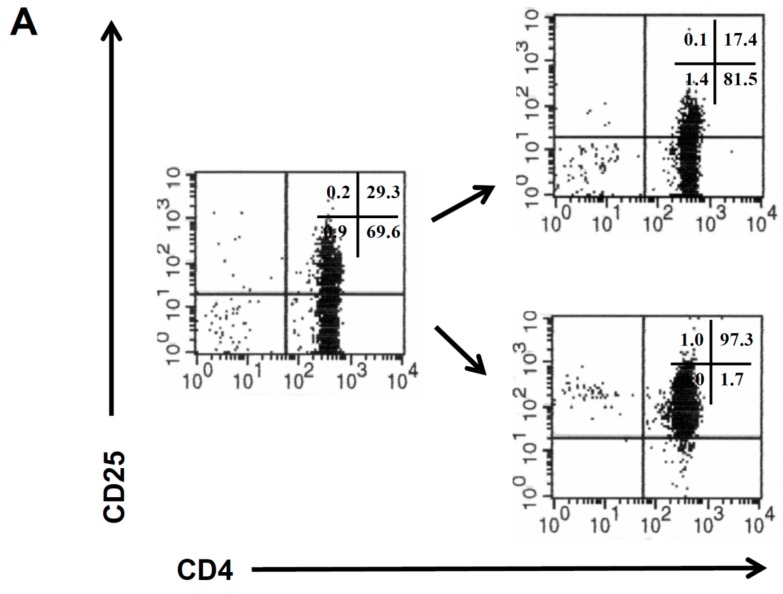

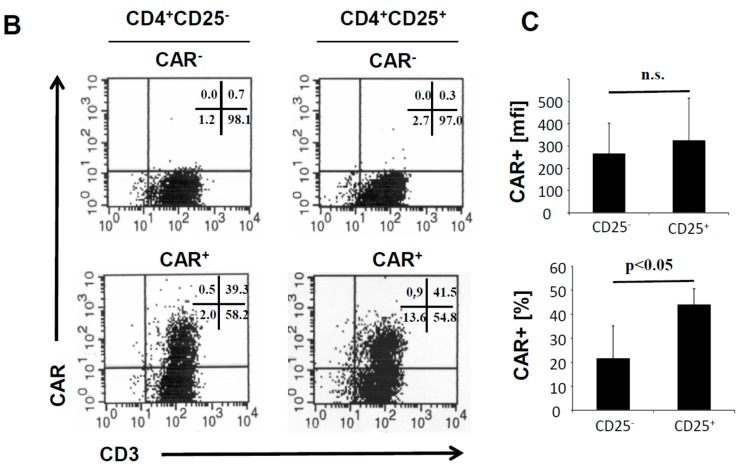
Isolation of class II−restricted CD4^+^ cytotoxic T cells (CD4^+^ T) cell subsets. (**A**) Peripheral blood lymphocytes were isolated from healthy donors and fractionated by magnetic cell sorting procedures into the CD4^+^CD25^−^ and CD4^+^CD25^+^ populations. Isolated cells were stained with anti-CD4 and anti-CD25 antibodies and analyzed by flow cytometry. (**B**,**C**) Expression of chimeric antigen receptors (CARs) in CD4^+^CD25^+^ and CD4^+^CD25^−^ T cells. Isolated CD4^+^CD25^−^ and CD4^+^CD25^+^ T cells were activated, expanded and engineered with an anti-carcinoembryonic antigen (CEA) CD28−ζ CAR as described in Materials and Methods. Modified cells were identified by staining with an anti-CD3 and anti-IgG antibody, which recognizes the extracellular IgG1 spacer within the CAR, and analyzed by flow cytometry. (**B**) Dot blots of a flow cytometric analysis of blood cells from a representative healthy donor. (**C**) Summary of mean fluorescence intensity (mfi) and number of CAR^+^ T cells in CD4^+^CD25^+^ and CD4^+^CD25^−^ T cells from healthy donors (*n =* 5). Numbers represent mean values ± Standard Deviation (SD). Significance was calculated by the Student’s *t* test.

CD4^+^CD25^−^ and CD4^+^CD25^+^ T cells were isolated from the peripheral blood of healthy donors by magnetic sorting procedures (Figure 1A), and the isolated CD4^+^ T cell populations were amplified as previously described [11]. CD4^+^CD25^−^ and CD4^+^CD25^+^ T cells from the same donor were retrovirally engineered with a CEA specific CAR which provides combined CD3ζ and CD28 signaling upon antigen encounter [10]. The CAR was expressed on similar levels on the cell surface of both T cell subsets (Figure 1B). The CAR transduction efficiency of CD4^+^CD25^+^ T cells was consistently higher than of CD4^+^CD25^−^ T cells (Figure 1C).

**Figure 2:**
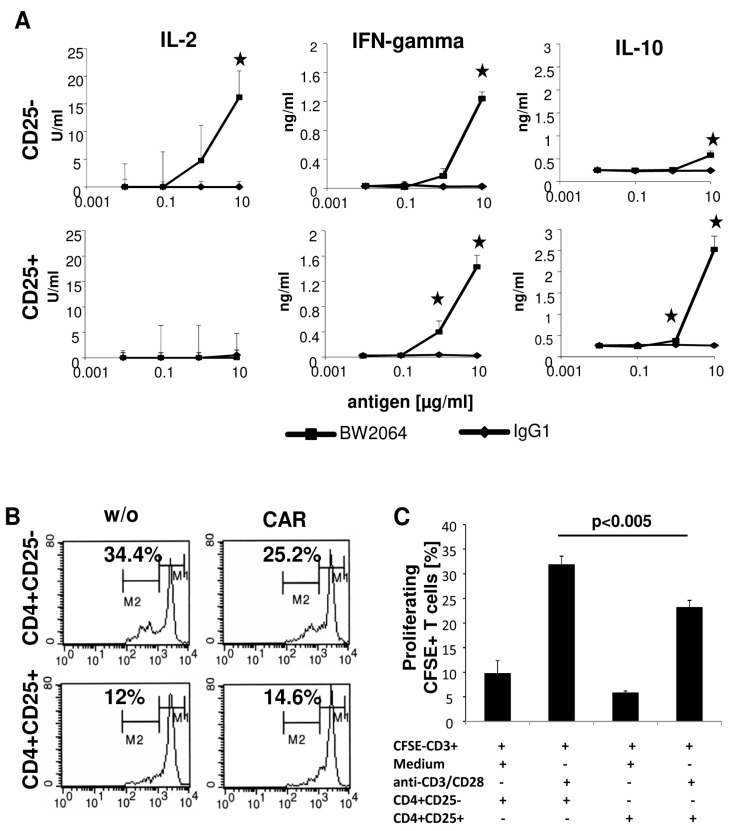
CD4^+^ T cells release a distinctive set of cytokines upon CAR mediated activation. (**A**) CD4^+^CD25^−^ and CD4^+^CD25^+^ anti-CEA CAR T cells (10^4^/well) were incubated in micro-titer plates coated with serial dilutions of the CAR specific anti-idiotypic monoclonal antibody (mAb) BW2064 or an IgG1 isotype control mAb (0.01–10 µg/mL) and cultivated for 48 h. Supernatants were recorded for cytokines by ELISA. Data represent the mean of technical replicates ± SD. Significant differences were calculated by the Student’s *t* test and significant data (*p* < 0.05) were indicated by asterisks. Representative results out of three experiments are shown. (**B**,**C**) Freshly isolated CD3^+^ T cells were labeled with CSFE and coincubated (5 × 10^4^ cells/well) either with non-labeled CD4^+^CD25^−^ and CD4^+^CD25^+^ CAR T cells (B,C), respectively, or non-modified T cells (w/o) (5 × 10^4^ cells/well) in the presence of the agonistic anti-CD3 mAb OKT3 (10 µg/mL) and anti-CD28 mAb 15E8 (1 µg/mL). After 5 days cells were recovered, pooled and CFSE-labeled cells were determined by flow cytometry (**B**). Cells of technical replicates were recovered and the numbers of cycling CFSE-labeled cells were recorded by flow cytometry (**C**). Numbers represent mean values ± SD. Significant differences were calculated by the Student’s t test. A representative experiment out of three is shown.

We activated the CAR CD4^+^ T cell subpopulations in an antigen-specific fashion by incubating with the solid phase bound anti-idiotypic mAb BW2064 which binds the CAR binding domain and acts as surrogate antigen [10]. T cell activation was monitored by recording IL-2, IFN-γ and IL-10 in the culture supernatants. CAR engineered CD4^+^CD25^−^ and CD4^+^CD25^+^ T cells released distinct patterns of cytokines; IL-2 was released exclusively by CD4^+^CD25^−^ CAR T cells, but not by CD4^+^CD25^+^ T cells, and IL-10 by CD4^+^CD25^+^ CAR T cells, while IFN-γ was secreted by both T cell subsets (Figure 2A). In particular, IL-10 and IFN-γ released by CAR engineered Treg cells indicated their anti-inflammatory capacity (Figure 2A). CD4^+^CD25^+^ T cells represent Treg cells since they suppressed the amplification of CSFE labeled CD3^+^ cells; CAR CD4^+^CD25^+^ Treg cells suppressed the amplification of CD3^+^ cells as did the CD4^+^CD25^+^ Treg cells without CAR (Figure 2B,C), indicating that genetic engineering did not alter the repressive capacities of Treg cells. The data are in line with our previously report [11] that ex vivo expansion of CD4^+^CD25^+^ Treg cells under these conditions preserve their phenotype and function.

**Figure 3:**
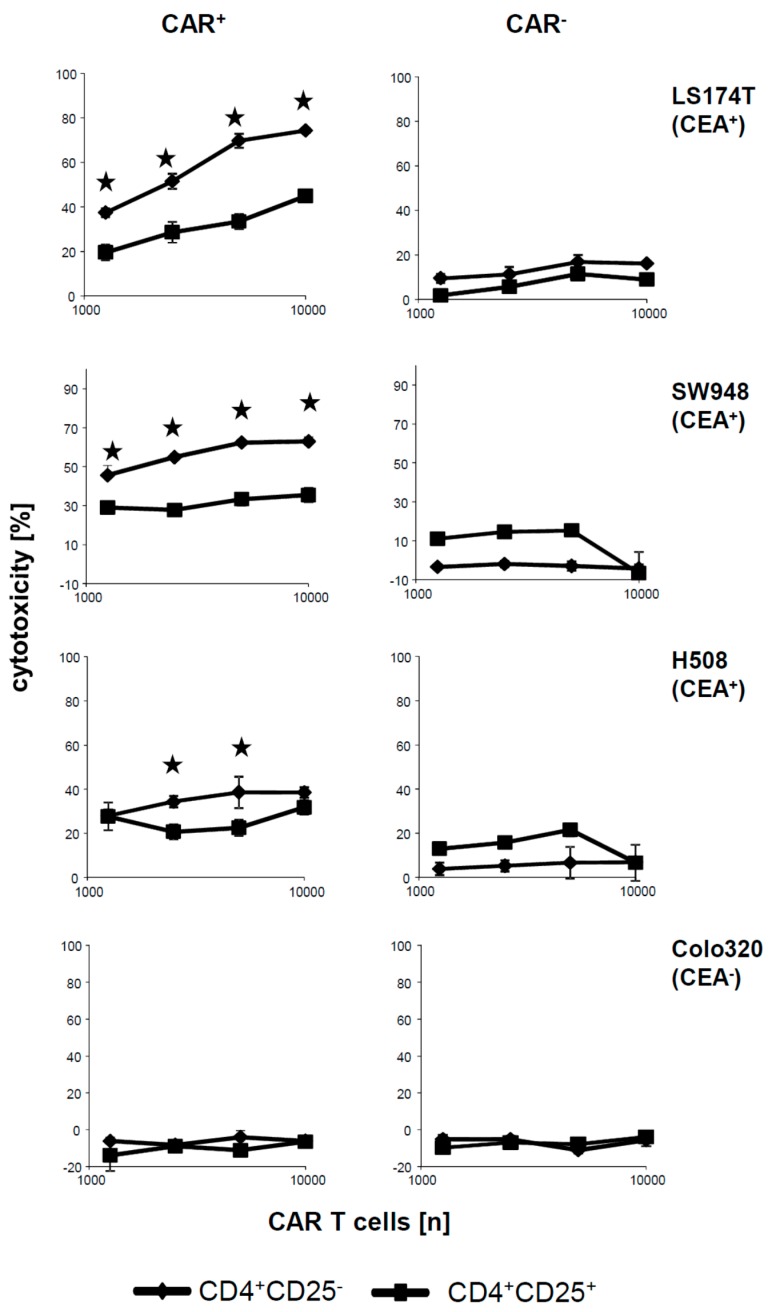
CD4^+^CD25^−^ and CD4^+^CD25^+^ CAR T cells represent CD4^+^ subpopulations with different lytic capabilities. CD4^+^ T cells were equipped with the anti-CEA CAR and CAR T cells (1.25–10 × 10^3^ CAR T cells/well) and non-modified T cells in same numbers were cocultivated for 48 h with CEA^+^ SW948, LS174T and H508 tumor cells, respectively, and for control with CEA^−^ Colo320 cells (each 2.5 × 10^4^ cells/well) in 96-well tissue culture plates. The viability of tumor cells was determined by the XTT assay and specific cytolysis calculated as described in Materials and Methods. Data represent mean of technical replicates ± SD. Significant differences were calculated by the Student’s *t* test and significant data (*p* < 0.05) were indicated by asterisks. Typical results out of three experiments are shown.

We asked whether the CAR engineered CD4^+^CD25^−^ T cells and CD4^+^CD25^+^ Treg cells have not only different cytokine responses but also different capabilities to lyse cognate target cells. To address the issue we co-incubated CD4^+^CD25^−^ and CD4^+^CD25^+^ CAR T cells with various CEA^+^ cell lines and recorded target cell lysis by an XTT based viability assay. As summarized in Figure 3, CD4^+^CD25^−^ CAR T cells lysed CEA^+^, LS174T, and SW948 tumor cells with high efficiencies. Lysis was antigen-specific and CAR dependent since CEA^−^ Colo320 cells were not lysed and the same T cells without CAR did not lyse CEA^+^ cells. In contrast, lysis of CEA^+^ cells by CAR Treg cells was substantially lower or at background levels as compared with CD4^+^CD25^−^ CAR T cells. In contrast to LS174T and SW948, tumor cells lysis of H508 tumor cells was low for both CD25^−^ and CD25^+^ CD4^+^ T cells, respectively. Since cytotoxicity of CAR Treg cells was poor towards all tested CEA^+^ cell lines, we concluded that the inability of cytolysis was mostly independent of the respective cell line, and rather an intrinsic property of the CD4^+^CD25^−^ CAR Treg cells.

**Figure 4:**
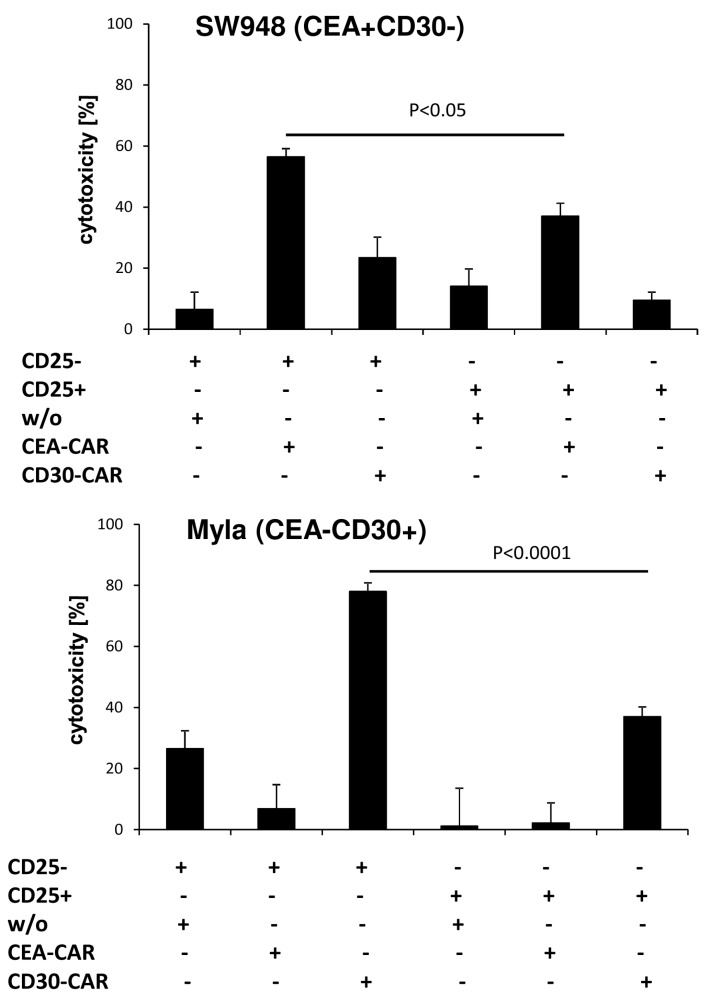
CD4^+^CD25^−^ and CD4^+^CD25^+^ CAR T cells represent subpopulations with different lytic capabilities independently of the CAR and target antigen. CD4^+^ T cells were equipped with the anti-CEA or anti-CD30 CAR and CAR T cells (2.5 × 10^4^ CAR T cells/well) and non-modified T cells in same numbers were cocultivated for 48 h with CEA^+^ CD30^−^ SW948 or CEA^−^CD30^+^ MyLa tumor cells (each 2.5 × 10^4^ cells/well) in 96-well tissue culture plates. The viability of tumor cells was determined by the XTT assay and specific cytolysis calculated. Data represent mean of technical replicates ± SD. Significant differences were calculated by the Student’s *t* test. A representative experiment out of three is shown.

To confirm the different cytolytic properties of CD4^+^CD25^−^ and CD4^+^CD25^+^ T cells, we engrafted the respective T cell subset with the anti-CEA and anti-CD30 CAR, respectively. Cytolysis was specifically driven by the CAR since CEA specific CD4^+^CD25^−^ CAR T cells lysed CEA^+^CD30^−^ SW948 target cells but not CEA^−^CD30^+^ MyLa cells; vice versa, anti-CD30 CD4^+^CD25^−^ CAR T cells lysed CEA^−^CD30^+^ MyLa cells but not CEA^+^CD30^−^ SW948 cells (Figure 4). While CAR triggered lysis by CD4^+^CD25^−^ CAR T cells was highly efficient, redirected cytolysis by CD4^+^CD25^+^ T cells was poor or at background levels. Since both CARs and target cells of different origin resulted in poor cytolysis, we concluded that the lack in killing capacities is an intrinsic property of CD4^+^CD25^+^ CAR T cells, and independent of the engrafted CAR or the target cell.

**Figure 5:**
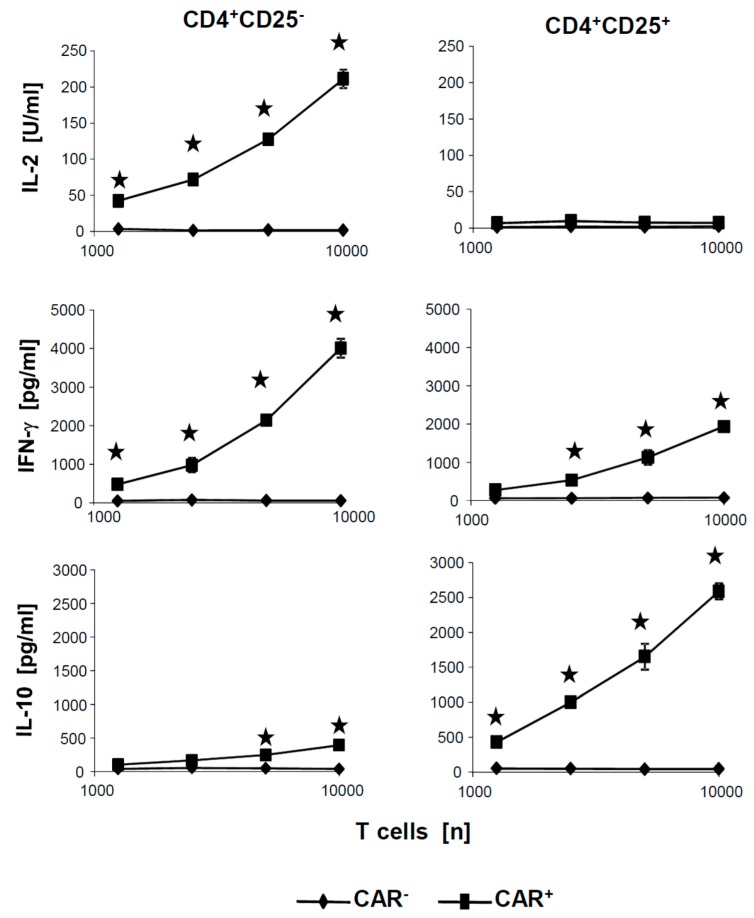
CD4^+^CD25^−^ and CD4^+^CD25^+^ T cells release a distinctive set of cytokines upon CAR engagement of target cells. CD4^+^ T cells with the anti-CEA CAR (1.25–10 × 10^3^ CAR T cells/well) and non-modified T cells in same numbers were cocultivated for 48 h in 96-well tissue culture plates together with CEA^+^ SW948 tumor cells (2.5 × 10^4^ cells/well). Supernatants were analyzed for cytokines by ELISA. Data represent mean of technical replicates ± SD. Representative results out of three experiments are shown. Significant differences were calculated by the Student’s *t* test and significant data (*p* < 0.05) were indicated by asterisks.

To exclude that the poor cytotoxicity of CD4^+^CD25^+^ CAR Treg cells was due to insufficient activation by the CAR, we tested the culture supernatants of the cytotoxicity assays for activation associated cytokines. Figure 5 exemplarily summarizes the levels of secreted cytokines after coincubation of CAR T cells with CEA^+^ SW948 target cells. IL-10 and IFN-γ were released at high levels by CAR Treg cells in a cell dose dependent fashion but no IL-2. Essentially, the same pattern of cytokines was recorded as upon CAR stimulation of the respective CAR T cells with the surrogate antigen BW2064 (cf. Figure 2). Taken together, we concluded that CD4^+^CD25^−^ and CD4^+^CD25^+^ CAR T cells were efficiently activated by the respective CAR, displayed a distinct pattern of secreted cytokines, however, showed substantially different cytolytic capacities upon CAR mediated activation.

**Figure 6:**
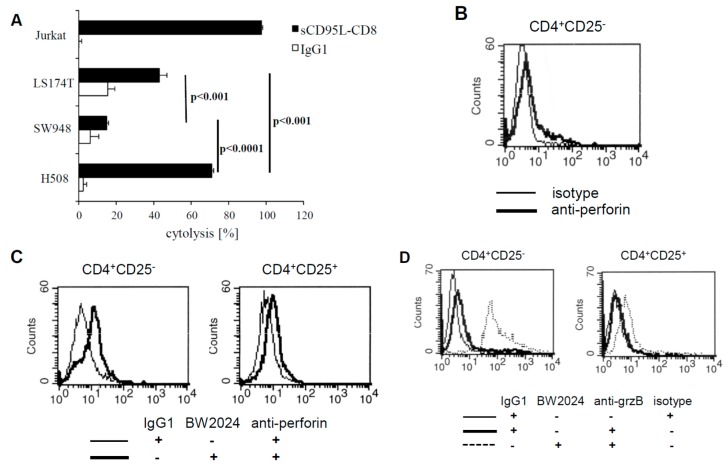
Susceptibility of target cells for FasL mediated cell death. (**A**) CEA^+^ LS174T, SW948 and H508 tumor cells and CEA^−^ Jurkat cells for control were incubated in 96-well micro-titer plates in the presence of recombinant CD8-Fas-L fusion protein (2 µg/mL) for 24 h. The viability of tumor cells was determined by the XTT assay and specific cytolysis calculated. Data represent mean of technical replicates + SD. Significant differences were calculated by the Student’s *t* test. A representative experiment out of three is shown. (**B**) Resting CD4^+^CD25^−^ T cells express low levels of perforin. CD4^+^CD25^−^ CAR T cells were cultured without stimulation for 48 h and tested by flow cytometry for intracellular perforin levels. An isotype matched mAb served as control for staining. (**C**,**D**) Perforin and granzyme B were upregulated in CD4^+^CD25^−^ but not in CD4^+^CD25^+^ CAR T cells upon CAR stimulation. CD4^+^CD25^−^ and CD4^+^CD25^+^ CAR T cells (2.5 × 10^4^/well) were stimulated in micro-titer plates coated with 10 µg/mL anti-idiotypic mAb BW2064 or with an isotype control IgG1 for 48 h. Cells from triplicates were pooled, stained with anti-perforin (**C**) or anti-granzyme B (**D**) mAbs, and recorded by intracellular flow cytometry.

There are two major pathways in mediating target cell lysis, i.e., (i) release of cytolytic effector proteins, in particular perforin and granzyme B, and (ii) Fas/FasL induced apoptosis. The latter mechanism can be displayed by CD4^+^CD25^+^ Treg cells, which is supported by the observation that Treg cells from cancer patients can induce Fas mediated apoptosis of susceptible target cells [20]. We therefore tested CEA^+^ cells, used as target cells for anti-CEA CAR Treg cells, for their susceptibility in Fas induced apoptosis by incubating with the recombinant FasL fusion protein. The highly FasL susceptible Jurkat cells served for control. As summarized in Figure 6A, the CEA^+^ cancer cells differ significantly in their sensitivity towards FasL induced apoptosis, ranging from resistance (SW948), to intermediate (LS174T), and to high susceptibility (H508). Notably, lysis of the CEA^+^ LS174T and SW948 cells by anti-CEA CAR CD4^+^ T cells did not correlate with their sensitivity towards FasL, making it unlikely that Fas mediated apoptosis is the major mechanism of killing. In particular, the highly Fas-sensitive H508 cell line was lysed with the lowest efficiency by CAR T cells, however, H508 cells were killed to some extent by CD4^+^CD25^+^ Treg cells, independently of the CAR. This is in line with the reported ability of Treg cells to induce apoptosis in a Fas-dependent pathway [21]. We concluded that Fas mediated apoptosis is an unlikely mechanism of killing by CAR CD4^+^ T cells. Our previous observation indicated that conventional CD4+ T cells can execute cytolysis by the release of granzyme and perforin [22]. We therefore recorded perforin and granzyme B expression of CD4^+^CD25^−^ and CD4^+^CD25^+^ CAR T cells, respectively, by flow cytometry. Without CAR stimulation, CD4^+^CD25^−^ CAR T cells expressed low amounts of perforin, which were upregulated upon CAR stimulation by solid phase bound antigen (Figure 6B,C). In contrast, CD4^+^CD25^+^ CAR Treg cells did not upregulate perforin upon CAR stimulation. Granzyme B was regulated in a similar fashion upon CAR activation, i.e., upregulated in CD4^+^CD25^−^ CAR T cells but not in CD4^+^CD25^+^ CAR Treg cells (Figure 6D). The data are in line with the recorded deficiency of CD4^+^CD25^+^ Treg cells in cytolysis as compared with the CD4^+^CD25^−^ T cells; the same properties were recorded upon CAR redirected activation clearly demonstrating a functional dichotomy between the two CD4^+^ T cell subsets with respect to cytolytic activities.

## 4. Discussion

The physiological role of cytotoxic CD4^+^ T cells is not completely understood, however, CD4^+^ T cells from the peripheral blood can efficiently be redirected for specific cytolysis by a CAR, which bypasses the MHC class II restriction. CAR engineered CD4^+^CD25^−^ T cells appear to be similarly efficient in cytolysis as the cytolytic CD8^+^ T cells when redirected by a CAR [22]. In contrast to CD8^+^ T cells, the majority of CD4^+^ T cells in the peripheral blood expresses low amounts of perforin and granzyme B, however, rapidly upregulate the cytolytic molecules upon T cell receptor (TCR) mediated activation [23,24]. Accordingly, stimulation through an engineered CAR induced upregulated perforin and granzyme B levels in CD4^+^CD25^−^ T cells implying that these cells are also capable of mediating cytolysis of target cells upon the appropriate CAR stimulation. Killing through Fas signaling is not the major mechanism since there is no correlation of the susceptibility for Fas mediated apoptosis with the CAR CD4^+^ T cell mediated cytotoxicity.

Apart thereof, CD4^+^CD25^+^ regulatory T cells in the peripheral blood were also reported to express perforin and granzyme B and to utilize these cytotoxic molecules to mediate immune suppression [9,25,26]. Upon bypassing MHC restrictions through an engineered CAR, CD4^+^CD25^+^ Treg cells were expected to mediate target cell lysis as do CD4^+^CD25^−^ T cells. However, our side-by-side analysis under thoroughly controlled conditions revealed that this is not the case; CAR CD4^+^CD25^+^ Treg cells showed poor cytolysis, if any, despite strong CAR activation as indicated by the release of high amounts of the suppressive cytokine IL-10. Poor cytolysis by the CAR Treg cells in the assays was mostly independent of the CAR, the target antigen and origin of target cells. The lack of cytolytic activities of CAR Treg cells is in line with the failure in upregulating the cytotoxic executor molecules upon CAR stimulation. Taken together, the data support our conclusion that cytolysis is not a major function of CD4^+^CD25^+^ Treg cells; strong CD28^−^CD3 stimulation by an engineered CAR did not induce significant killing by Treg cells although the Treg cells were strongly activated.

Our data have major implications for the CAR T cell therapy using CD4^+^ T cells; CD4^+^CD25^−^ cells exhibit high cytotoxic properties upon CAR engagement of antigen, as do conventional cytotoxic CD8^+^ T cells resulting in redirected lysis of the targeted cells. As CD8^+^ T cells the CAR redirected CD4^+^CD25^−^ T cells can be likewise used as killers in the therapy of cancer in order to eliminate the cognate cancer cells. While CAR T cells are predominantly used in an autologous setting, the adoptive transfer of allogeneic CAR T cells may also be envisaged. In this context we expect that the allogeneic CD4^+^CD25^−^ CAR T cells will have a low risk of TCR mediated toxicity due to their MHC class II restriction, however, will trigger cytolytic activites towards CAR targeted cells. On the other hand, cell therapy with CAR engineered Treg cells provides benefits in the treatment of chronic inflammatory diseases by mediating immune repression upon CAR triggered activation without redirected cytotoxicity. This is of particular interest since CAR Treg cells showed superior in suppressing immune reactions when compared with non-modified Treg cells in pre-clinical models [15,16,17]. The low cytolytic capability of Treg cells moreover suggests that their application in an allogeneic setting using allo CAR Treg cells without major risk of inducing a cytolytic graft-versus-host attack. In conclusion, CAR modified Treg cells are suggested to be suitable for auto- or allogeneic cell therapy of autoimmune diseases due to their immune repression without lytic activities.
